# Identification of Novel Immunologic Checkpoint Gene Prognostic Markers for Ovarian Cancer

**DOI:** 10.1155/2022/8570882

**Published:** 2022-09-15

**Authors:** Xiao Huo, Xi Zhang, Shuhong Li, Shuzhen Wang, Hengzi Sun, Mo Yang

**Affiliations:** ^1^Center of Basic Medical Research, Institute of Medical Innovation and Research, Cancer Center, Peking University Third Hospital, Beijing, China PCODE: 100191; ^2^Department of Obstetrics and Gynecology, Peking University Third Hospital, Beijing, China PCODE: 100191; ^3^Department of Obstetrics and Gynecology, Beijing Chao-Yang Hospital, Capital Medical University, Beijing, China PCODE: 100025; ^4^Medical Center for Human Reproduction, Beijing Chao-Yang Hospital, Capital Medical University, Beijing, China PCODE: 100025

## Abstract

Ovarian cancer has a higher resistance to chemotherapy, displaying the highest mortality rate among gynecological cancers. Recently, immune checkpoint inhibitor therapy is an effective treatment for selected patients. However, a low response rate for immune checkpoint treatment was observed for ovarian cancer patients. Therefore, it is necessary to identify ovarian cancer patients who might gain benefits from immune checkpoint treatment. Datasets containing ovarian cancer samples with mRNA-seq and clinical follow-up data were downloaded from different databases like The Cancer Genome Atlas (TCGA) and Gene Expression Omnibus (GEO). The researchers applied the univariate analysis for selecting the immune checkpoint genes (ICGs) at a significance level of *P* < 0.05 as the candidate ICGs. The Spearman correlation coefficients were calculated to compare the correlation between tumor mutation burden and candidate ICGs, and the Kaplan-Meier plots were generated. They also assessed the external validation datasets and the results of immunohistochemical staining. 46 and 35 ICGs were extracted from the TCGA and GEO datasets, respectively, and we categorized the ICGs into 3 expression patterns. Nine (TCGA) and three (GEO) ICGs were significantly related to the prognosis. Univariate survival analysis indicated a significant prognostic relationship between the expression levels of ICOS, TIGIT, and TNFRSF8 and overall survival (OS). Moreover, the expression of ICOS and TIGIT also presented a significantly positive relationship with the CD8A expression. Importantly, patients with a higher CD8A and ICOS expression level (ICOS-H/CD8A-H) showed a better survival rate compared to other patients. Stratified analysis using TIGIT, TNFRSF8, and CD8A expression also showed an improved prognosis for the high TIGIT/high CD8A expression subgroup and the low TNFRSF8/low CD8A expression subgroup compared to the other subgroups. This study identified different immune subtypes that can predict the OS of ovarian cancer patients. This data could prove to be beneficial for making important clinical decisions and designing individual immunotherapeutic strategies.

## 1. Introduction

With rapid population growth and aging across the world, cancer is anticipated to become a primary cause of death and a major impediment to improving the life expectancy of people in the next few decades [1]. Ovarian cancer shows the highest mortality rate among the different types of gynecological cancers. According to GLOBOCAN estimates, ovarian cancer was responsible for ≈295,414 new cases and caused 184,799 related deaths worldwide in 2018. In an earlier study, the researchers determined that the 5-year survival rate of ovarian cancer patients was around 22-30% [2]. Platinum-based systemic chemotherapy is the main method for treating ovarian cancer. The vast majority of patients eventually die due to resistance to chemotherapy [3]. Therefore, in this study, the researchers have focused on screening for novel markers and viable treatment targets for ovarian cancer.

The growth and progression of tumors are related to immunosuppression [4]. It is known that cancer cells can activate different immune checkpoint pathways with immunosuppressive functions, thereby suppressing the activity of the immune system to achieve recognition and elimination of immune cells [5]. Immune checkpoint therapy can enhance the antitumor immune response by blocking this suppressive signal from the immune system [6–8]. Recent reports show that patients with multiple malignancies have benefited from immune checkpoint inhibitor therapy [9–11]. However, there is a low response rate for immune checkpoint treatment in ovarian cancer [12–14]. In many patients with ovarian cancer, immunotherapy has no significant clinical benefit, but some patients have shown a favorable response, which indicated that the immune checkpoint therapy has a specific selectivity for certain patient populations [15, 16]. Thus, it becomes important to differentiate between various immune subtypes of ovarian cancer for improving the design of the clinical trials and identifying the patients who could gain benefit from the immune checkpoint treatment.

In this study, the researchers analyzed the gene expression of 47 immune checkpoint genes (ICGs) and determined the relationship between their expression and patient prognosis. Then, they integrated the somatic mutation data for determining the relationship between immunotherapy biomarkers like the tumor mutation burden (TMB), neoantigen expression, and ICG (PD1/PD-L1, CTLA4, etc.) expression, which were widely used as the biomarkers for immunotherapy response. Finally, they studied the relationship between the expression levels of ICGs and the signature genes associated with immune activation to understand the correlation between the ovarian cancer immune subtypes and patient prognosis.

## 2. Materials and Methods

### 2.1. Sources and Preprocessing of Data

#### 2.1.1. The Cancer Genome Atlas (TCGA) Data

The researchers used the TCGA (http://cancergenome.nih.gov) Genomic Data Commons-Application Programming Interface (GDC API) for downloading all the data related to ovarian cancer RNA-seq data for 360 samples. They also downloaded the clinical follow-up information of the patients on 14^th^ December, 2018. They preprocessed the data as follows: (1) the data samples without any follow-up clinical data or an overall survival (OS) duration <30 days were eliminated from the analysis. (2) They removed the data related to normal tissue samples. (3) They removed the data corresponding to genes with a fragment per kilobase of exon model per million reads mapped (FPKM) = 0, in >50% of the samples.

#### 2.1.2. Gene Expression Omnibus (GEO) Data

The researchers downloaded the GSE26712 expression data in MINiML format from the NCBI database (https://www.ncbi.nlm.nih.gov/geo/query/acc.cgi?acc=GSE26712). The GSE26712 contained 183 samples with clinical characteristics. The following steps were used for preprocessing the GSE26712 data: (1) only primary tumor data were retained, while all information regarding the normal tissue samples was eliminated. (2) The researchers carried out the unified conversion of the OS data (years or months) into days. (3) They mapped each chip probe to the human gene symbols using the bioconductor package. (4) They retained only the expression profiles of the immune-related genes. (5) Data were filtered according to the above TCGA data preprocessing process. After preprocessing, the number of samples in the GSE26712 dataset was 183. Detailed information on the TCGA and GEO datasets has been presented in Table 1. In addition, 47 ICGs are shown in Supplementary S1.

### 2.2. Univariate Analysis

The researchers conducted the univariate analysis using the R package survival, and all ICGs having a significance level of *P* < 0.05 were chosen as the candidate ICGs.

### 2.3. Survival Analysis

The Kaplan-Meier (KM) survival analysis using the survival package was performed to estimate the associations between the expression levels of the prognosis-related genes and overall survival.

### 2.4. Genomic Mutation Analysis

According to the mutation annotation file (MAF) predicted by TCGAmutect2, the intron interval was removed and the silent mutations were annotated; then, the number of mutations in each sample was counted. Finally, TMB was calculated as follows: TMB = total number of mutations in each sample/total exon length. Those mutations were exon mutation.

### 2.5. Statistical Analysis

The main immunogenomics methods were integrated, the data related to the immune tumor microenvironment (TME) for 33 cancers were analyzed by TCGA, and the total lymphocyte infiltration rate was assessed to predict neoantigens. The Spearman correlation coefficients were calculated for comparing the correlation between the TMB and candidate ICGs, and the Kaplan-Meier plots were generated using R3.4.3. The data was considered to be significant at *P* < 0.05.

### 2.6. Immunohistochemical (IHC) Staining

Here, the researchers collected the data regarding 208 human epithelial ovarian cancer samples, with their corresponding clinical follow-up information, for the period duration ranging from 2009 to 2015, from the Department of Obstetrics and Gynecology, Pathology of Peking Union Medical College Hospital. All patients who were included in this study shared the following characteristics: (1) suffered from epithelial ovarian cancer, (2) had undergone cytoreductive surgery and additional chemotherapy, and (3) possessed follow-up information. On the other hand, the samples were eliminated based on the following exclusion criteria: (1) ovarian sex cord-stromal tumor, ovarian germ cell tumor, or metastatic cancer, (2) received unstandardized treatment, (3) did not offer their informed consent, (4) did not receive follow-up visits, and (5) had inadequate pathological samples.

The researchers conducted the follow-up procedure until January 2020. All pathological diagnoses were verified by a pathologist. The researchers sought approval for this project from the Ethical Committee (Peking Union Medical College Hospital). They also acquired informed consent from the patients or their family members. The researchers carried out IHC as described earlier [17, 18]. They used the following antibodies: CD8A (1 : 200, Abcam ab93278), CD30 (1 : 500, Abcam ab134080), TIGIT (1 : 200, Abcam ab243903), and ICOS (1 : 300, Abcam ab224644). They graded the intensity of the IHC as follows: 1+ ~4+ (weakest to strongest among all samples). The area of the positive cancer cells in each microscopic field was classified in the following manner: 1+ ~4+ (<25 to >75% for all samples). They acquired the total score (ranging from 5 to 80) after multiplying the two scores by one another and multiplying the value by 5. If the total score ranged between 0 and 42, it was regarded as “low expression”, while the score ranging between 43 and 80 was considered as “high expression”. Two pathologists independently reviewed the slides based on their individual criteria, and then they used the consensus interpretation as the final interpretation. In the case of a discrepant interpretation, a third pathologist was consulted to resolve the disagreements.

## 3. Results

### 3.1. Relationship between ICGs and Ovarian Cancer Prognosis

**Figure 1:**
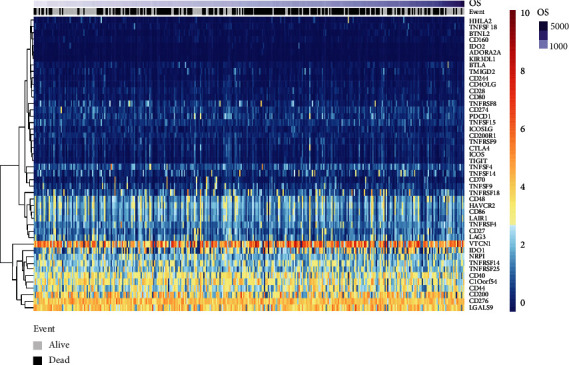
Heatmap of the ICG expression level based on the data derived from the TCGA dataset. High-expression group: red; Medium-expression group: green; Low-expression group: blue.

To determine candidate ICGs, the researchers extracted the data regarding 46 ICGs with expression in the TCGA dataset.  Figure 1 presents the gene expression levels of ICGs. Based on their expression levels, the researchers categorized the 46 ICGs into 3 different expression groups, i.e., high-expression (red), medium-expression (green), and low-expression (blue) groups. The genes included in the high-expression group were VTCN1, CD200, CD276, and LGALS9, as they showed a higher expression level in all the cancer samples. On the other hand, the medium-expression group included TNFRSF18, TNFRSF4, CD27, and LAG3 and their expression levels varied significantly among the different cancer samples. In addition, the genes included in the low-expression group were IDO2, ADORA2A, and KIR3DL1, as they showed a lower expression level in the majority of the cancer samples.

**Figure 2:**
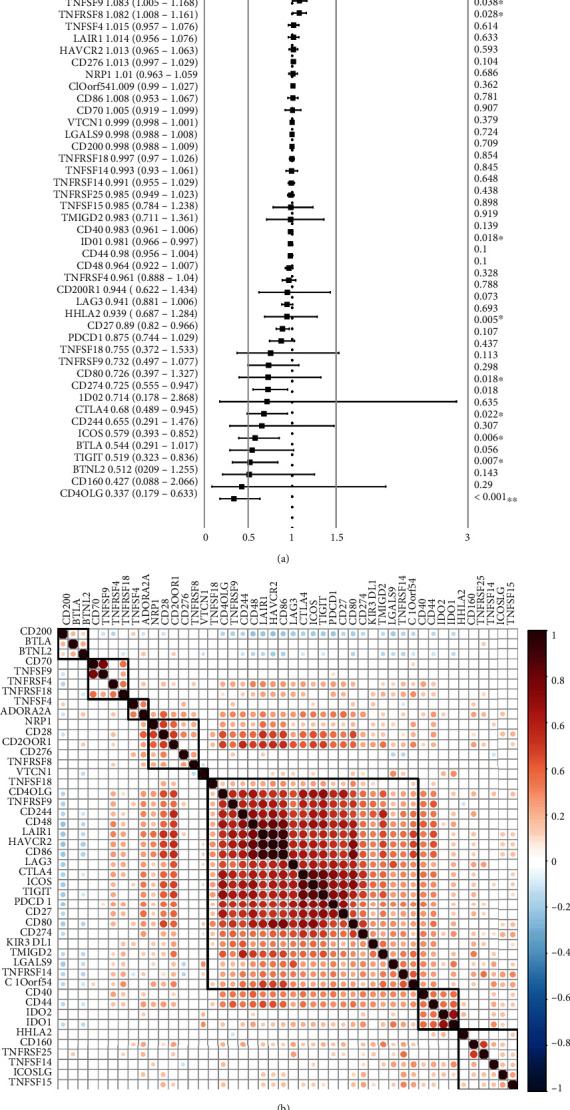
Expression of the ICGs. (a) Relationship between the ICG expression and patient prognosis. (b) Correlation of the ICG expression levels. Note: here, the researchers have only presented the data regarding the gene pairs showing a significant correlation; blank denotes that no significance was observed according to the correlation test results.

The researchers used the univariate Cox regression analysis for determining the relationship between 46 expressed ICGs and the prognosis of patients with ovarian cancer. They identified 9 ICGs that were significantly related to prognosis (Figure 2(a), log − rank *P* < 0.05). Among them, PD-L1 (CD274) and IDO1 showed a significant correlation with a good prognosis. These two genes are also important biomarkers for immunotherapy response, so it has been speculated that the effect of immunotherapy on ovarian cancer is significant. However, the correlation analysis of the ICGs expressions showed that these ICGs were positively correlated to each other and displayed a significant aggregation effect (Figure 2(b)). These results highlighted a cooperative expression relationship between the ICGs.

The researchers performed the same analysis on the GSE26712 dataset (which included 35 ICGs with differential expression). The expression patterns of these ICGs were clearly clustered into 3 different categories (Supplementary S2A). The genes in the three groups from the GSE26712 analysis and the genes of the three groups from the TCGA analysis showed a high consistency; VTCN1, IDO1, TNFRSF25, CD40, CD200, and LGALS9 showed high expression levels in both the datasets. TNFRSF18, CD48, CD86, TNFRSF4, CD27, and LAG3 showed a moderate expression level in both the datasets, while HHLA2, CD160, ICOS, TNFSF15, and CD28 showed a low expression level in the 2 datasets. Although NRP1 and TNFRSF14 were identified as having high expression levels in the TCGA dataset and showed medium expression levels in the GSE26712 dataset, the overall expression level of NRP1 and TNFRSF14 was observed to be moderate and varied widely across different samples. The researchers carried out the univariate survival analysis for determining the relationship between the expression levels of 35 ICGs and OS and noted that 3 ICGs were significantly related to prognosis with regards to the OS (Supplementary S2B). In the GSE26712 dataset, the researchers noted that the expression levels of ICGs were positively related to each other. The observations also indicated the presence of a significant aggregation effect (Supplementary S2C) that was consistent with the results of the TCGA dataset.

### 3.2. Relationship between Different ICGs and Immune Checkpoint Treatment Biomarkers

**Figure 3:**
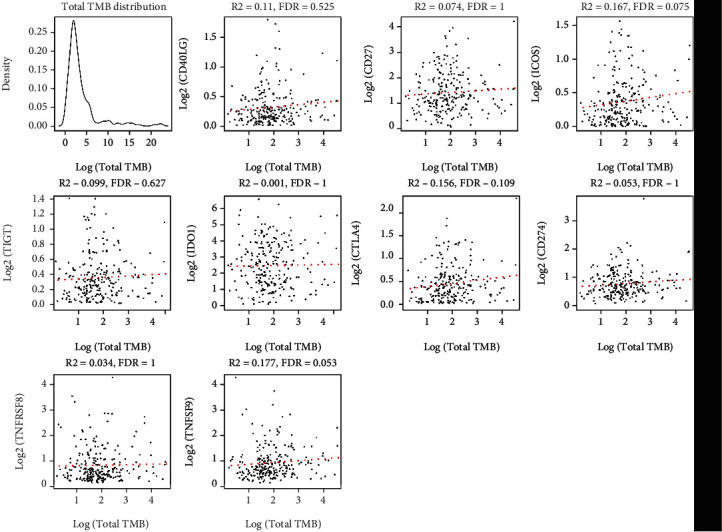
Scatter plot depicting the ICG expression level and TMB. *R^2^*: correlation coefficient; FDR: false discovery rate.

The anticancer effect of immunotherapy mainly depends on the recognition of cancer cell-specific antigens by immune cells. Therefore, compared to those with few genetic mutations, the cancer patients with more genetic mutations experience enhanced antigen production by cancer cells, which are then recognized by the body's immune cells. Thus, the higher the tumor mutational burden (TMB) in the tumor tissues, the higher the probability that the patient gains benefit from immunotherapy [19]. Firstly, based on the somatic mutation data derived from the TCGA dataset, the researchers calculated the TMB of each sample. Secondly, they determined the expression levels of the 9 ICGs, which showed a significant relationship with patient prognosis (OS). They also assessed the link between the TMB and the 9 ICGs with the help of the Spearman technique (TMB distribution was nonnormal, Shapiro test *P* < 1*e* − 5). However, no correlation was observed between the TMB and expression levels of the nine ICGs (Figure 3).

**Figure 4:**
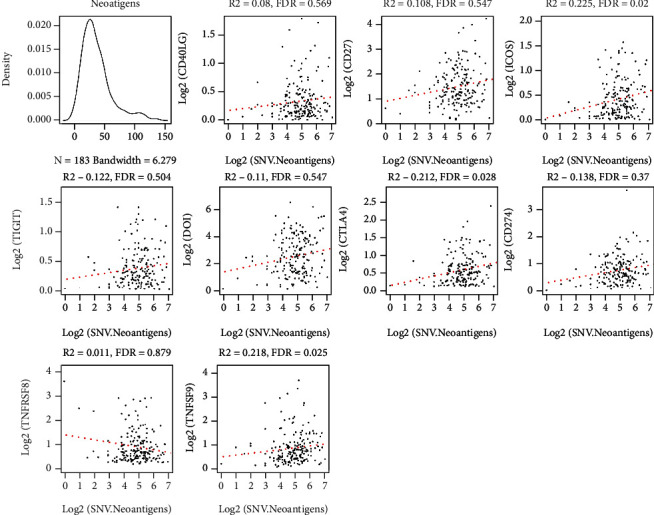
Scatter plot depicting the ICG expression level and neoantigen levels. *R^2^*: correlation coefficient; FDR: false discovery rate.

Tumor somatic mutations, when present in protein-coding genes, lead to the production of abnormal proteins (neoantigens) that are presented by the major histocompatibility complex (MHC) class I. These novel peptides bind to the T cell receptor (TCR) and induce an adaptive immune response against cancer. The researchers used the somatic mutation data derived from the TCGA dataset and analyzed the relationship between the ICG expression levels and neoantigens (Figure 4). The results showed that the expression of ICOS, CTLA4, and TNFSF9 genes was significantly and positively related to the level of neoantigens (R2 > 0.2 and false discovery rate (FDR) < 0.05). ICOS and CTLA4 are factors for the benign prognosis of OS. They speculated that the high expression of ICOS and CTLA4 corresponded to the high neoantigen levels, while a generally high level of neoantigen corresponds to a high level of TMB, which is speculated to be appropriate for the immune checkpoint inhibitor therapy.

**Figure 5:**
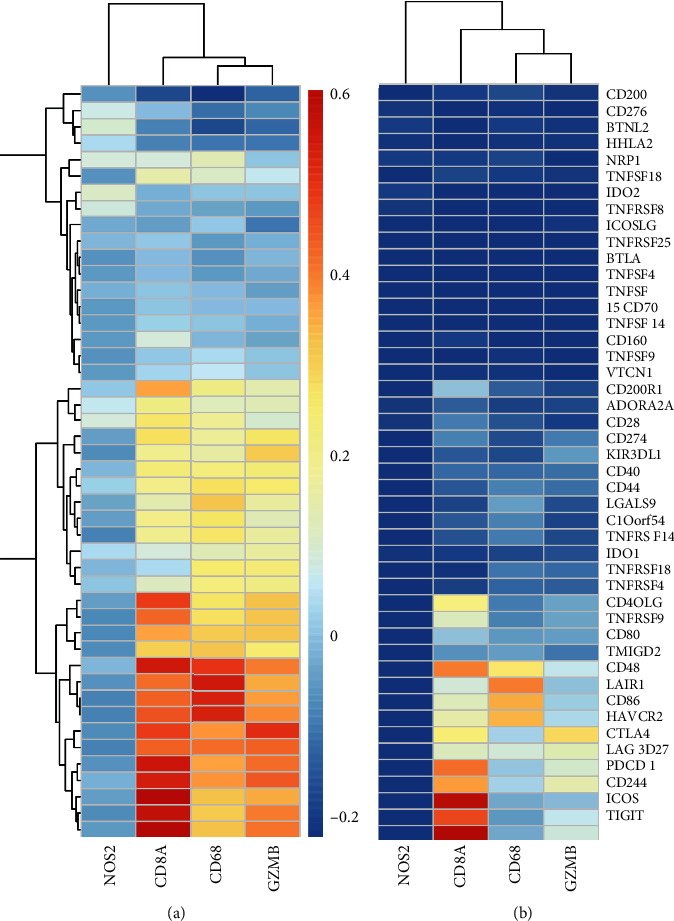
Correlation between the ICG expression level and immune resistance pathway. (a) Heatmap depicting the correlation coefficient between ICGs and genes involved in the adaptive immune resistance pathway. (b) *P* value of the correlation coefficient test conducted between ICGs and the genes involved in the adaptive immune resistance pathway. The *P* value undergoes −log10 conversion.

CD8^+^ T cells can produce Interferon-gamma (IFN*γ*) that upregulates the expression of adaptive immune resistance pathway genes (like the PD-1/PD-L1 axis and IDO1). Hence, the researchers analyzed the correlation between the expression level of genes (CD68, GZMB, CD8A, and NOS2) involved in the adaptive immune resistance pathway, which was obtained from MSigDB and ICGs. The results showed that the expression levels of the genes involved in the adaptive immune resistance pathway, except NOS2, showed a high positive correlation with the ICG expression (Figure 5(a)). The significance tests of correlation coefficients revealed that the majority of the correlations between these genes were extremely significant (*P* < 1*e* − 5, Figure 5(b)). With regards to the data downloaded from the GSE26712 dataset, the researchers analyzed the relationship between the expression levels of the genes involved in the adaptive immune resistance pathway and ICGs and noted that they were mainly positively correlated. The results also showed that the CD8A, CD86, and CD48 genes showed a significantly positive correlation (Supplementary S3). This was consistent with the results for the TCGA dataset.

### 3.3. Association between ICG Expression-Defined Ovarian Cancer Subtypes and Prognosis

**Figure 6:**
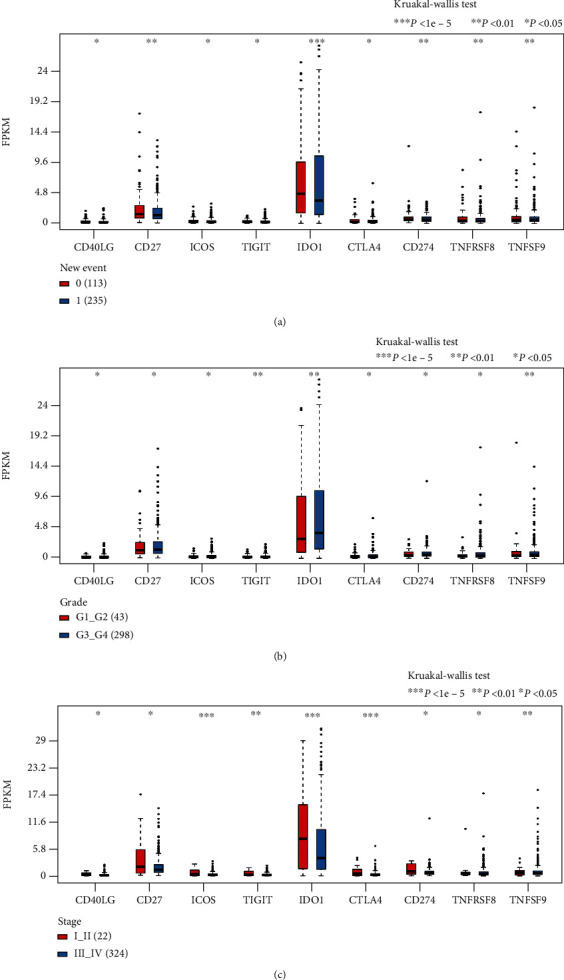
Expression levels of the 9 ICGs (FPKM). (a) Nine ICGs were differentially expressed when the samples were stratified based on the neoantigen level. (b) Nine ICGs were differentially expressed when the samples were stratified based on grade. (c) Nine ICGs were differentially expressed when the samples were stratified based on stage. ∗*P* < 0.05, ∗∗*P* < 0.01, ∗∗∗*P* < 1*e* − 5.

After combining all the clinical data derived from the TCGA dataset, the researchers evaluated the relationship between 9 ICGs that showed a significant correlation between OS and clinical characteristics. They primarily focused on analyzing the expression levels of the 9 ICGs based on grade, stage, and new events. The 9 ICGs were categorized into the high-expression and low-expression groups (Supplementary S4). The statistical tests indicated that the CD274 gene expression showed significant differences when the samples were stratified by stage (rank test *P* < 0.05); thus, the CD274 expression level in the advanced tumor samples was significantly decreased compared to that in the early tumor samples (Figure 6(a)–6(c)).

ICOS, TIGIT, and TNFRSF8 are important immune checkpoint genes. The univariate survival analysis revealed a significant prognostic relationship between ICOS, TIGIT, and TNFRSF8 expression and OS in TCGA dataset. Through the previous analysis, the researchers observed that the expression of the ICOS and TIGIT genes was significantly and positively related to the CD8A expression, which further indicated that CD8A led to the upregulation of the genes involved in the adaptive immune resistance pathway.

**Figure 7:**
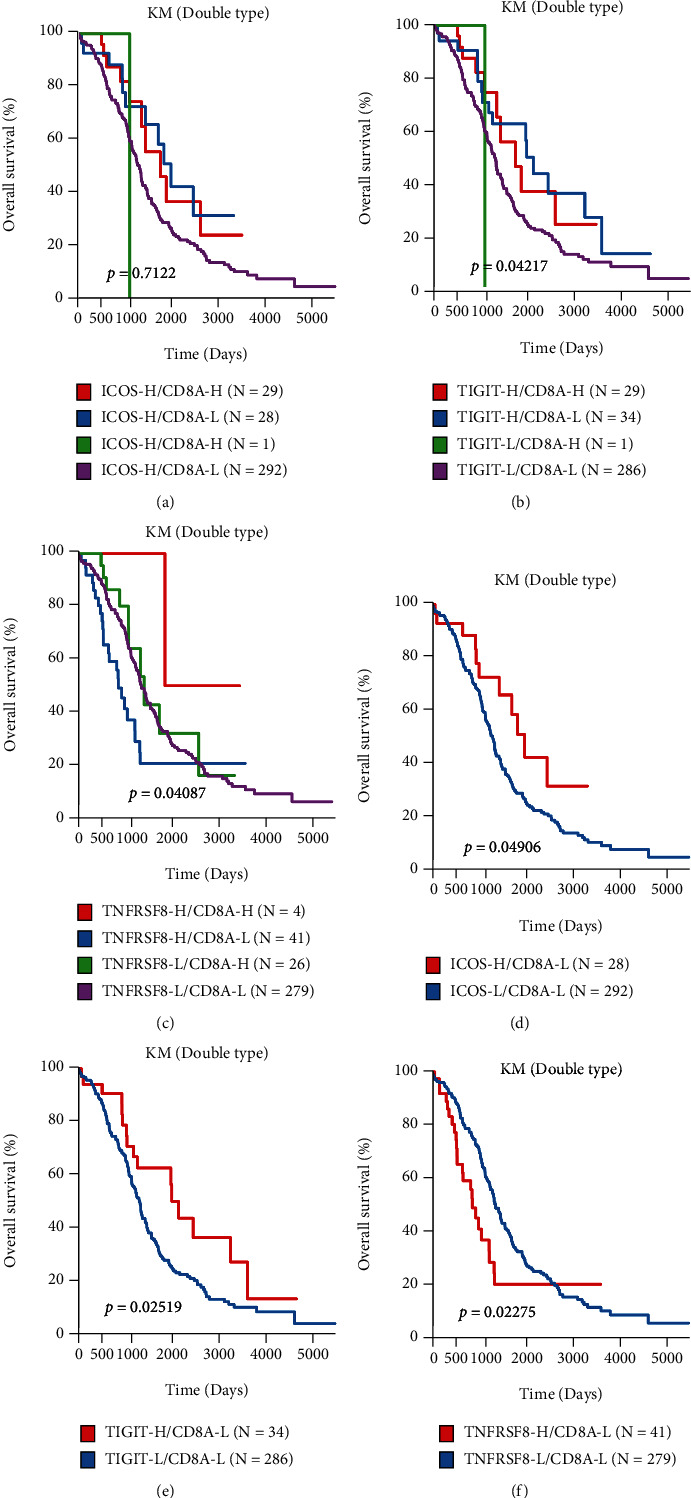
The Kaplan-Meier survival curve of different groups. (a, d) ICOS and CD8A; (b, e) TIGIT and CD8A; (c, f) TNFRSF8 and CD8A.

As the expression level of the ICOS, TIGIT, TNFRSF8, and CD8A genes were decreased in the ovarian cancer samples (Figure 1, Supplementary S2), the researchers categorized patients into the high-expression (H) and low-expression (L) groups based on the density distribution of the gene expression levels (Supplementary S5), considering the deviation from the first main distribution interval (i.e., density peak) as a threshold (red vertical line in Supplementary S5) for categorizing the genes into the high- (H-) and low- (L-) expression groups. After incorporating the H-/L- expression groups of ICOS, TIGIT, CD8A, and TNFRSF8, the researchers categorized the ovarian cancer samples into 4 groups. Survival analysis of the 3 pairs of gene classification samples indicated the presence of significant differences in the OS across the 4 sample types. The group having a higher CD8A, ICOS (ICOS-H/CD8A-H), and TIGIT (TIGIT-H/CS8A-H) expression level displayed the best prognosis. On the other hand, the group having a lower CD8A, ICOS (ICOS-H/CD8A-H), and TIGIT (TIGIT-H/CS8A-H) expression level displayed the worst prognosis (Figure 7(a)–7(b)). For the TNFRSF8 gene, the combination of lower CD8A and higher TNFRSF8 expression level showed the worst prognosis, while the combination of a higher CD8A and lower TNFRSF8 expression level showed the best prognosis (Figure 7(c)). In this study, the researchers used 2 types of samples with the best and worst prognosis for analyzing the OS, independently. They noted a significant difference across the samples in prognosis (Figure 7(d)–7(f)). In the GSE26712 dataset, due to the lack of high expression of all three pairs of genes, the researchers could not observe the corresponding prognostic information (Supplementary S6).

They also obtained the data related to the 6 immune subtypes reported in an earlier study [20] and extracted the molecular subtype data of these samples for comparison. They observed that the ICOS-H/CD8A-L, TIGIT-H/CD8A-L, and TNFRSF8-H/CD8A-L subtypes were primarily composed of the C1, C2, and C4 subtypes, whereas ICOS-L/CD8A-L, TIGIT-L/CD8A-L, and TNFRSF8 -L/CD8A-LIS4 subtype was mainly composed of C1, C2, C3, C4. Compared with ICOS-L/CD8A-L, TIGIT-L/CD8A-L, and TNFRSF8-L/CD8A-LIS4 group, ICOS-H/CD8A-L, TIGIT-H/CD8A-L, and TNFRSF8-H/CD8A-L have a higher proportion of C1 samples and a lower proportion of C2 samples. Among them, C1 showed a worse prognosis, which is consistent with the results noted in this study. The two subtypes the researchers reported can be used as a supplement to the six immune subtypes in the previous study (Supplementary S7).

The researchers obtained the expression profile dataset of PD-L1 drug-treated patients with metastatic urothelial carcinoma from the previous study [21] and extracted the expression profiles of the TNFRSF8, CD8A, and TIGIT genes. The researchers used the multivariate regression technique for scoring the risk of patients and categorized them into high-risk and low-risk groups. The researchers noted that patients in the high-risk group showed a significantly worse prognosis, as shown in Supplementary S8A. They also analyzed the risk scores of various PD-L1 response states and observed that the PD scores were significantly higher than other groups, as shown in Supplementary S8B. These results indicate that TNFRSF8, CD8A, and TIGIT gene expression levels are closely related to immunotherapy, and they may be potential markers of immunotherapy.

In this study, the researchers collected the data from serous carcinoma patients. Their results indicated that the expression levels of ICGs were positively correlated. They also noted a significant aggregation effect, as shown in Figure 3(c). In addition, the researchers retrieved the dataset GSE63885 with different histological types from the GEO database. The original data of the chip that they downloaded was standardized using RMA, and the expression profile of ICGs was extracted. It contains a total of 45 ICGs genes, 9 clear cell ovarian cancer cases, 12 endometrioid ovarian cancer cases, 73 serous ovarian cancer cases, and 7 undifferentiated ovarian cancer cases. The researchers, respectively, calculated the expression correlation between the ICGs of the four histological types of ovarian cancer patients, as shown in Supplementary S9, and noted that the correlation was mainly positive. They also analyzed the expression difference between cancer and normal samples in the GSE26712 cohort, as shown in Supplementary S10, and observed that there are 24 (66.7%) genes that are significantly different.

### 3.4. ICG Expression Defines the Subtype of Ovarian Cancer according to IHC

**Figure 8:**
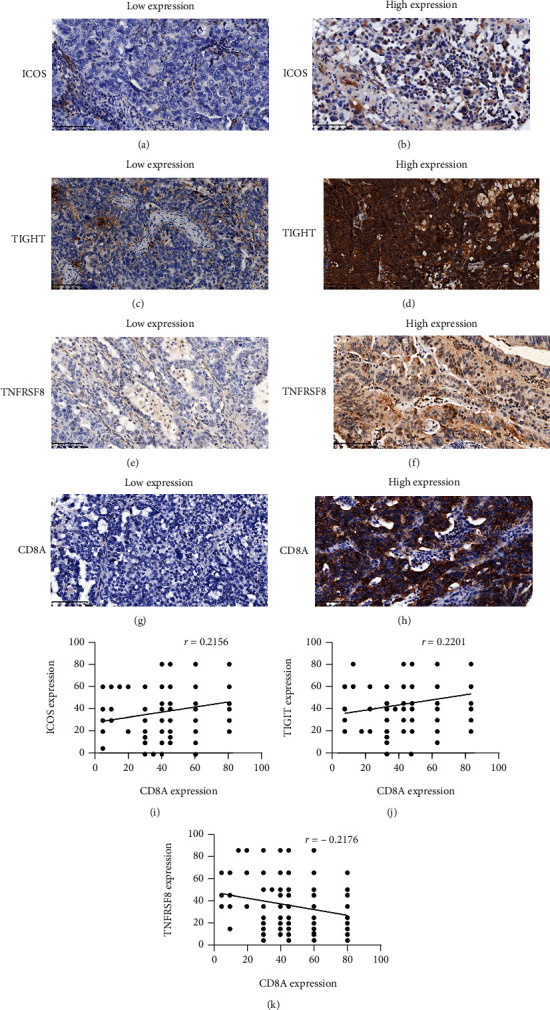
Immunohistochemistry for ICOS, TIGIT, TNFRSF8, and CD8A. Ovarian cancer samples (*n* = 208). Weak immunostaining scores for ICOS (a), TIGIT (c), TNFRSF8 (e), and CD8A (g) and strong immunostaining scores for ICOS (b), TIGIT (d), TNFRSF8 (f), and CD8A (h). The relative expression of the ICOS, TIGIT, TNFRSF8, and CD8A genes is depicted (i, j, k) (200×).

To evaluate the results on the basis of the TCGA dataset, the researchers verified the relationship between the ICOS, TIGIT, and TNFRSF8 expression levels and patient prognosis, along with the favorable survival of the subgroup with a higher CD8A level with high ICOS/TIGIT or low TNFRSF8 expression level, by analyzing the protein expression in an independent cohort of ovarian cancer patients from Peking Union Medical College Hospital (*n* = 208) (Figure 8(a)–8(h)). Table 1 presents a summary of the clinicopathologic characteristics of 208 ovarian cancer patients.

**Figure 9:**
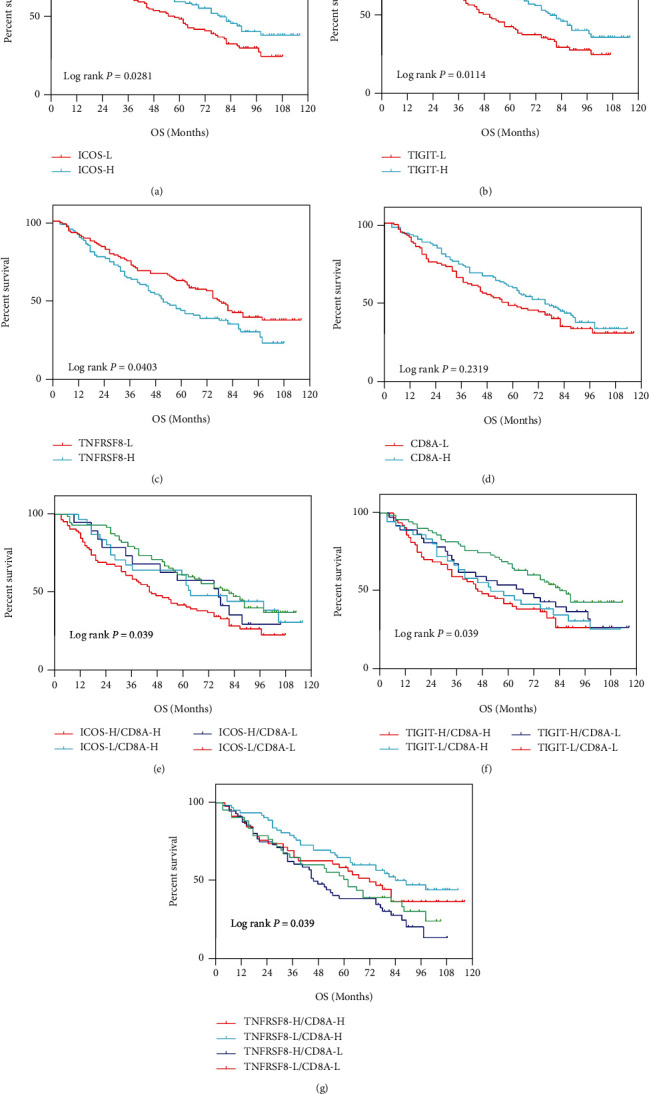
Overall survival (OS) curves depicted for the ovarian cancer patients based on the gene expression levels of ICOS (a), TIGIT (b), TNFRSF8 (c), CD8A (d), ICOS/CD8A (e), TIGIT/CD8A (f), and TNFRSF8/CD8A (g). Samples were stratified according to the median expression value for each gene.

No significant association between the expression levels of ICOS, TIGIT, TNFRSF8, and CD8A and the clinical tumor characteristics like the pathologic stage or histologic grade (*P* > 0.05 for all data samples) was observed. In line with the TCGA data, the researchers noted a positive correlation between the ICOS and TIGIT gene expression and the CD8A expression; whereas, the TNFRSF8 expression showed a negative correlation with the CD8A expression (Figure 8(i)–8(k)). Patients having a higher ICOS (OS, HR = 0.6840, 95% CI 0.4844-0.9563, *P* = 0.028) and TIGIT (OS, HR = 0.6490, 95% CI 0.4537-0.9009, *P* = 0.011) expression level in the IHC analysis showed a better survival rate compared to the patients having a lower ICOS or TIGIT expression level. The results also indicated that the patients with a high TNFRSF8 expression level showed a lower survival rate (OS, HR = 1.419, 95% CI 1.020-2.029, *P* = 0.040) (Figure 9(a)–9(d)).

Moreover, the researchers verified their results that indicated that higher CD8A expression in the high expression of ICOS was related to an improved survival rate among ovarian cancer patients. The researchers categorized the samples into four groups: ICOS-H/CD8A-H, ICOS-L/CD8A-H, ICOS-H/CD8A-L, and ICOS^−^L/CD8A-L. The data showed that the patients with a higher CD8A+ ICOS expression (ICOS-H/CD8A-H) level showed the best survival rate, while patients belonging to the ICOS-L/CD8A-H and ICOS-H/CD8A-L groups showed a similar survival rate. The ICOS^−^L/CD8A-L group had the worst survival (*P* = 0.039, Figure 9(e)). Stratified analysis using TIGIT, TNFRSF8, and CD8A expression also showed improved prognosis for the TIGIT-H/CD8A-H (*P* = 0.032) and TNFRSF8-L/CD8A-L subgroups (*P* = 0.019; Figures 9(f) and 9(g)).

## 4. Discussion

Ovarian cancer is one of the deadliest gynecological tumors, and its risk of occurrence during a woman's lifetime is ≈1-2% [22]. Although surgical treatments and platinum-based chemotherapy have definitely improved the prognosis of affected patients, many of the patients still die owing to the recurrence of the tumors and platinum resistance; the ovarian cancer patients at advanced stages show a 5-year survival rate of only 20-30% [23]. Recently, targeted drugs such as bevacizumab and olaparib have been used for BRCA-mutated ovarian cancer. Unfortunately, there has been limited survival improvement in patients with recurrent ovarian cancer. New and effective treatment strategies are needed to overcome drug resistance in ovarian cancer and prevent tumor progression [24–26].

In recent years, with the in-depth study of immune regulatory molecules in the immune system and tumor microenvironment, tumor immunotherapy has become a popular topic in clinical research. The most extensive clinical studies are immune checkpoint inhibitors, such as those targeting cytotoxic T lymphocyte-associated protein 4 (CTLA-4) and the programmed cell death receptor protein-1 (PD-1), as well as its ligand (PD ligand 1 or PD-L1), have been applied in the clinical treatment of a variety of tumors [27–30]. Most immune checkpoint inhibitor-based treatment strategies involve a combination with chemotherapy or targeted therapy for first-line treatment for ovarian cancer or for recurrent ovarian cancer. During the phase 1 clinical trial (NCT01772004) of the PD-L1 inhibitor, avelumab, the researchers noted that 125 patients with the relapsed or refractory epithelial ovarian cancer showed a 1-year progression-free survival rate of 10.2%. When they were administered the avelumab monotherapy, their OS duration was 11.2 months [31]. The clinical data of KEYNOTE-028 ovarian cancer indicated that the overall response rate (ORR) of the 26 PD-L1-positive epithelial ovarian cancer patients who received pembrolizumab treatment was 11.5%, while their median progression-free survival and OS values were recorded to be 1.9 months and 13.1 months, respectively [32]. Phase 2 clinical trial results of the PD-1 inhibitor, nivolumab, that was administered to the platinum-resistant relapsed ovarian cancer patients indicated an ORR of 15%, while their median progression-free survival and OS values were recorded to be 3.5 months and 20 months, respectively [33]. Based on the results of an earlier study, the ORR of immune checkpoint inhibitors in relapsed or resistant ovarian cancer ranges between 10 and 15%. Therefore, it is imperative to perform immune subgroup analysis to improve clinical efficacy in ovarian cancer patients.

At present, the major barrier to optimal cancer immunotherapy that can be used for treating ovarian cancer patients is shown to be the immunosuppressive TME, which is shaped by the intrinsic biologic characteristics of this tumor. Many cancer systems have offered evidence that showed that the T-cell expression of the inhibitory immune checkpoint receptors is an effective mechanism that could be used by the tumor cells to inhibit or evade the host immunity. Developing biomarkers that could be used as an effective strategy for predicting the response of the patients to various immunotherapies is necessary, thus facilitating the selection of the appropriate treatments based on the available results. Furthermore, these biomarkers could enable the researchers to rationally combine the different immunotherapies, which could help in minimizing their toxicities.

Currently, although numerous molecular subtyping has been developed in ovarian cancer, such as IFNG, CD30, CXCL13, PRF1 GBP1, and ETV7 CTLA-4, they provided limited prognostic information or are not validated in the clinical samples [34–36]. Therefore, beyond these pathways, additional tolerogenic mechanisms could be investigated in ovarian cancer therapy, as their relevance in the development of novel combination therapies can be explored. The more potential immune checkpoint gene should be explored in ovarian cancer, which also can facilitate the genomic analyses of the genotype immunophenotype relationships that help in deepening the understanding of the immunogenomic profile of ovarian cancer [37].

In this study, the researchers have attempted to identify the immune subgroup of ovarian cancer based on the expression profile of ICGs and to explore the clinical significance of the expression profile by assessing its correlation with survival. Firstly, they used the TCGA and GEO data for analyzing the 47 ICGs. According to their expression levels, the ICGs were categorized into the high-, medium-, and low-expression groups. Correlation analysis indicated the presence of a synergistic relationship between ICGs. Prognostic analysis of OS yielded 9 ICGs that showed a significant correlation with the prognosis. Secondly, after combining the TMB and neoantigen levels, the researchers observed a significant positive relationship between the expression levels of favorable prognostic genes, like ICOS and CTLA4, and levels of neoantigens (R2 > 0.2 and FDR < 0.05). It was speculated that a high expression level of genes like ICOS and CTLA4 corresponds to a higher neoantigen level, while a higher neoantigen level was linked to a higher TMB value that is especially observed in the patients who can be considered to be suitable for receiving treatment using immune checkpoint inhibitors. Correlation analysis of the expression of the genes involved in the adaptive immune pathway (CD8A, CD68, GZMB, and NOS2) and ICGs revealed that, except for NOS2, the other three genes showed a significantly positive association with a majority of the ICGs, which indicated that the adaptive immune pathway genes could significantly affect the ICG expression and exert a regulatory effect. Finally, the significance test indicated that the expression pattern of the CD274 gene has significant differences when ovarian cancer patients were stratified by stage (rank test *P* < 0.05). In other words, the CD274 expression level in advanced tumor samples was significantly lower compared to that in the early tumor samples. By integrating the expression levels of ICOS, TIGIT, TNFRSF8, and CD8A, ovarian cancer patients can be divided into 4 subgroups, which have different prognosis. Future work will focus on the effectiveness of relevant immune checkpoint inhibitors for ovarian cancer.

ICOS expression has been described by infiltrating and proliferating tumor-associated Tregs in breast cancer, which is correlated with poor prognosis [38]. In ovarian cancer, Toker et al. recently demonstrated that tumor infiltrating Tregs display an increased expression of ICOS, higher than in melanoma [39]. TIGIT blockade or TIGIT deletion promotes NK cell-mediated antitumor reactivity in vitro and in vivo [40]. CD8A encodes part of cell surface glycoprotein on most cytotoxic T lymphocyte, which includes adaptive immune response-induced CD8+ cytolytic T cells, and plays a crucial role in the antitumor activity of anti-PD-L1 [41, 42]. Those data indicated the reliability of molecular subtype classification.

The present study has some limitations. First, the clinical information downloaded from the TCGA databases was limited and incomplete. Second, the prediction subgroups need further validation in multicenter, large-scale clinical trials and prospective studies.

## 5. Conclusion

In conclusion, using TCGA and GEO data, the researchers analyzed the expression levels of 47 ICGs and their relationships with patient prognosis. Secondly, they analyzed the correlation between the expression levels of the ICGs (PD1/PD-L1, CTLA4, etc.), biomarkers that could serve as the immunotherapy response indicators, and other biomarkers. Finally, the expression levels of ICOS, TIGIT, TNFRSF8, and CD8A were integrated for the purpose of grouping ovarian cancer samples by prognosis. Our findings provide a potential opportunity for the development of immunotherapeutic strategies based on the immune subtypes of ovarian cancer.

## Figures and Tables

**Table 1:** Characteristics of the ovarian cancer patients.

Characteristics	No.	%
Age (y), median (range)	54 (25-75)	100%
Stage		
I–II	52	25.0%
III	112	53.8%
IV	44	21.2%
Histological subtype		
Serous	152	73.1%
Endometroid	18	8.7%
Clear cell	17	8.2%
Mucinous	21	10.1%
Differentiation grade		
G1	47	22.6%
G2	45	21.6%
G3	77	37.0%
Unknown	39	18.8%
Residual disease		
Optimal	124	59.6%
Suboptimal	48	23.1%
Unknown	36	17.3%

## Data Availability

The datasets used and/or analyzed during the current study are available from the corresponding authors on reasonable request.

## Supplementary Materials

Supplementary Materials Supplementary S1. the name of 47 immune checkpoint genes. Supplementary S2. A: ICGs express heat map on the GSE26712 data set. Red: high-expression group;, green: medium-expression group; blue: low-expression group. B: The relationship between the expression of ICGs and the prognosis; C: The correlation between the expression level of ICGs. Note: Only the gene pairs with a significant correlation test are displayed, and a blank indicates that the correlation test is not significant. Supplementary S3. the expression relationship between the adaptive immune resistance pathway genes and ICGs. Supplementary S4. The groups of these 9 ICGs. Supplementary S5. the high-expression (H) group and low-expression (L) group according to the density distribution of these gene expression levels. Supplementary S6. the corresponding prognostic information. Supplementary S7. The comparation of different molecular subtypes of ovarian cancer. Supplementary S8. The selected genes predict patient with immune therapy treatment. Supplementary S9. The expression correlation between the ICGs of the four histological types of ovarian cancer patients. Supplementary S10. The expression of selected genes in normal tissue.
