# Supplementary figures and images for: Identification of Novel Immunologic Checkpoint Gene Prognostic Markers for Ovarian Cancer
Source: J Oncol. 2022 Sep 15;2022:8570882. doi: 10.1155/2022/8570882 (PMC9499758; doi:10.1155/2022/8570882)

Supplementary S10. The expression of selected genes in normal tissue.


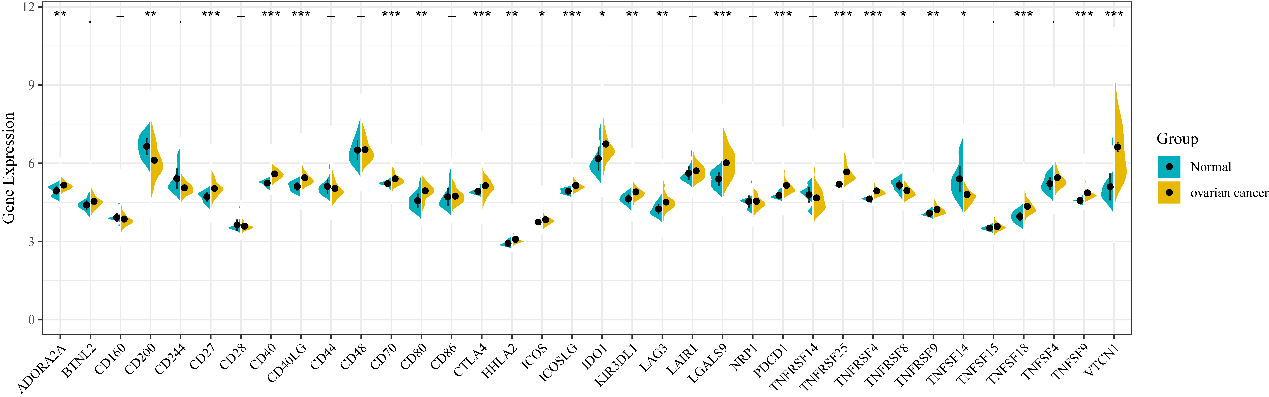

Supplement: Supplementary Materials — Supplementary S1. the name of 47 immune checkpoint genes. Supplementary S2. A: ICGs express heat map on the GSE26712 data set. Red: high-expression group;, green: medium-expression group; blue: low-expression group. B: The relationship between the expression of ICGs and the prognosis; C: The correlation between the expression level of ICGs. Note: Only the gene pairs with a significant correlation test are displayed, and a blank indicates that the correlation test is not significant. Supplementary S3. the expression relationship between the adaptive immune resistance pathway genes and ICGs. Supplementary S4. The groups of these 9 ICGs. Supplementary S5. the high-expression (H) group and low-expression (L) group according to the density distribution of these gene expression levels. Supplementary S6. the corresponding prognostic information. Supplementary S7. The comparation of different molecular subtypes of ovarian cancer. Supplementary S8. The selected genes predict patient with immune therapy treatment. Supplementary S9. The expression correlation between the ICGs of the four histological types of ovarian cancer patients. Supplementary S10. The expression of selected genes in normal tissue. [file 8570882.f1.zip › Supplementary S10.docx]

Supplementary S4. The groups of these 9 ICGs.


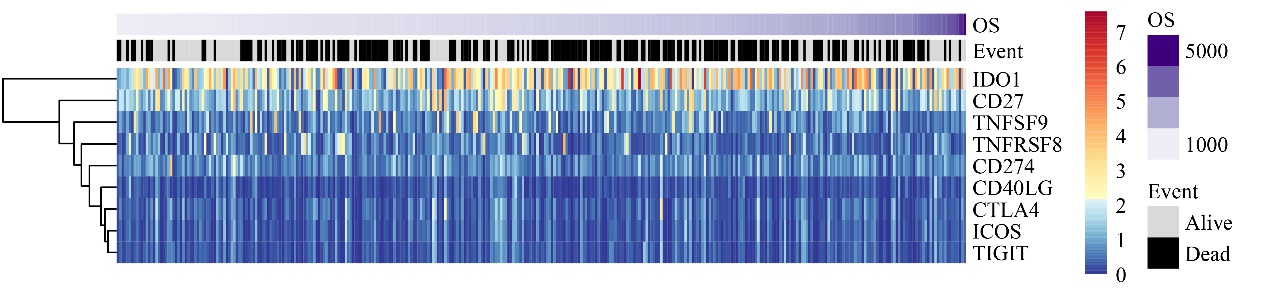

Supplement: Supplementary Materials — Supplementary S1. the name of 47 immune checkpoint genes. Supplementary S2. A: ICGs express heat map on the GSE26712 data set. Red: high-expression group;, green: medium-expression group; blue: low-expression group. B: The relationship between the expression of ICGs and the prognosis; C: The correlation between the expression level of ICGs. Note: Only the gene pairs with a significant correlation test are displayed, and a blank indicates that the correlation test is not significant. Supplementary S3. the expression relationship between the adaptive immune resistance pathway genes and ICGs. Supplementary S4. The groups of these 9 ICGs. Supplementary S5. the high-expression (H) group and low-expression (L) group according to the density distribution of these gene expression levels. Supplementary S6. the corresponding prognostic information. Supplementary S7. The comparation of different molecular subtypes of ovarian cancer. Supplementary S8. The selected genes predict patient with immune therapy treatment. Supplementary S9. The expression correlation between the ICGs of the four histological types of ovarian cancer patients. Supplementary S10. The expression of selected genes in normal tissue. [file 8570882.f1.zip › Supplementary S4.docx]

Supplementary S6. the corresponding prognostic information


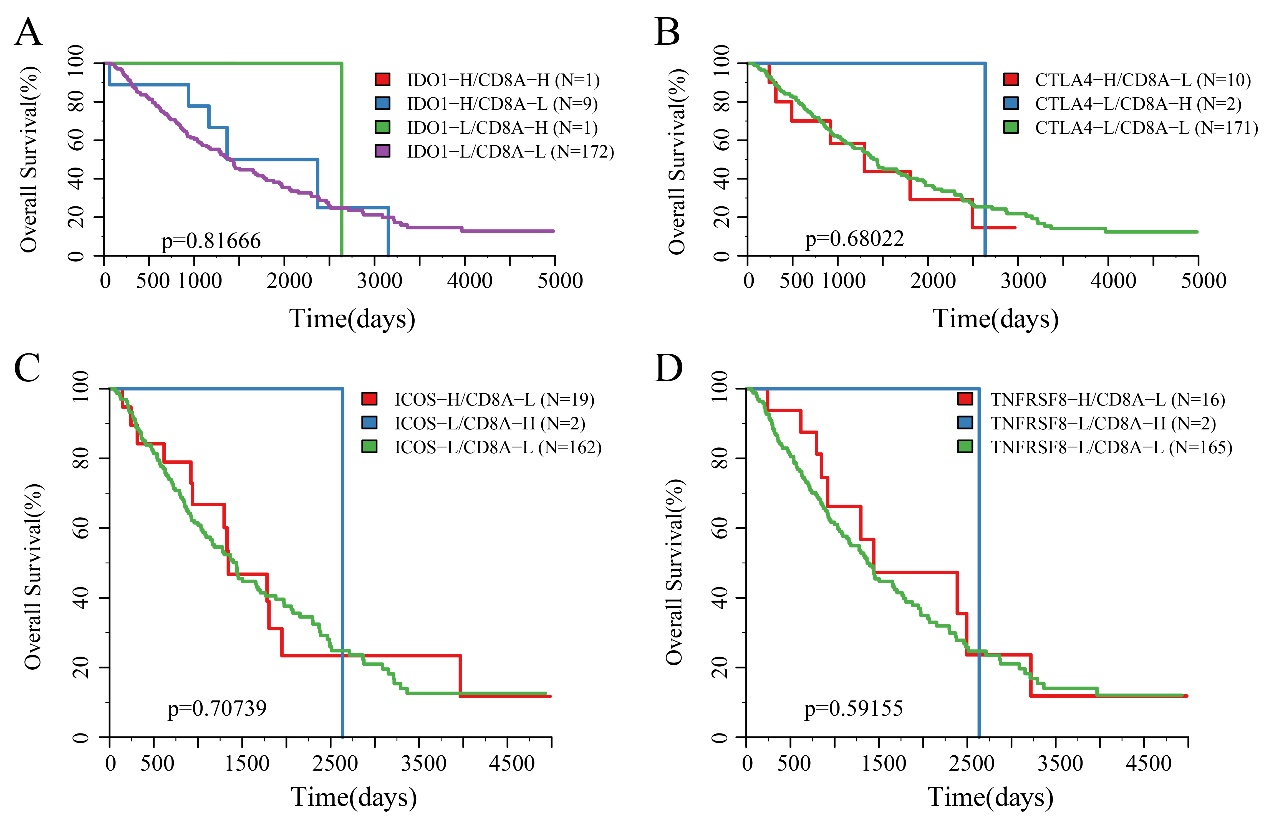

Supplement: Supplementary Materials — Supplementary S1. the name of 47 immune checkpoint genes. Supplementary S2. A: ICGs express heat map on the GSE26712 data set. Red: high-expression group;, green: medium-expression group; blue: low-expression group. B: The relationship between the expression of ICGs and the prognosis; C: The correlation between the expression level of ICGs. Note: Only the gene pairs with a significant correlation test are displayed, and a blank indicates that the correlation test is not significant. Supplementary S3. the expression relationship between the adaptive immune resistance pathway genes and ICGs. Supplementary S4. The groups of these 9 ICGs. Supplementary S5. the high-expression (H) group and low-expression (L) group according to the density distribution of these gene expression levels. Supplementary S6. the corresponding prognostic information. Supplementary S7. The comparation of different molecular subtypes of ovarian cancer. Supplementary S8. The selected genes predict patient with immune therapy treatment. Supplementary S9. The expression correlation between the ICGs of the four histological types of ovarian cancer patients. Supplementary S10. The expression of selected genes in normal tissue. [file 8570882.f1.zip › Supplementary S6.docx]
